# Risk Factors for Extended Duration of Acute Diarrhea in Young Children

**DOI:** 10.1371/journal.pone.0036436

**Published:** 2012-05-08

**Authors:** Tor A. Strand, Pushpa R. Sharma, Håkon K. Gjessing, Manjeswori Ulak, Ram K. Chandyo, Ramesh K. Adhikari, Halvor Sommerfelt

**Affiliations:** 1 Centre for International Health, University of Bergen, Bergen, Norway; 2 Medical Microbiology, Innlandet Hospital Trust, Lillehammer, Norway; 3 Division of Infectious Disease Control, Norwegian Institute of Public Health, Oslo, Norway; 4 Department of Child Health, Tribhuvan University, Kathmandu, Nepal; 5 Department of Epidemiology, Norwegian Institute of Public Health, Oslo, Norway; 6 Department of Public Health and Primary Health Care, University of Bergen, Bergen, Norway; Aga Khan University, Pakistan

## Abstract

**Objective and Background:**

We sought to identify predictors of extended duration of diarrhea in young children, which contributes substantially to the nearly 1 1/2 million annual diarrheal deaths globally.

**Methods:**

We followed 6-35 month old Nepalese children enrolled in the placebo-arm of a randomized controlled trial with 391 episodes of acute diarrhea from the day they were diagnosed until cessation of the episode. Using multiple logistic regression analysis, we identified independent risk factors for having diarrhea for more than 7 days after diagnosis.

**Results:**

Infants had a 17 (95% CI 3.5, 83)-fold and toddlers (12 to 23 month olds) a 9.9 (95% CI 2.1, 47)-fold higher odds of having such illness duration compared to the older children. Not being breastfed was associated with a 9.3 (95% CI 2.4, 35.7)-fold increase in the odds for this outcome. The odds also increased with increasing stool frequency. Furthermore, having diarrhea in the monsoon season also increased the risk of prolonged illness.

**Conclusion:**

We found that high stool frequency, not being breastfed, young age and acquiring diarrhea in the rainy season were risk factors for prolonged diarrhea. In populations such as ours, breastfeeding may be the most important modifiable risk factor for extended duration of diarrhea.

## Introduction

Approximately 1.3 million children die of diarrhea annually [1] and it is one of the leading causes of death and illness in children of developing countries. Efforts to reduce diarrheal morbidity and mortality have concentrated on prompt hydration therapy and have reduced the mortality from acute dehydrating diarrhea [2]. The introduction of rotavirus vaccines will further reduce the number of children dying from acute diarrhea.

Diarrhea may be classified into three syndromes, dysentery, acute diarrhea, and diarrhea with extended duration, where episodes compromising child nutrition are the most dangerous. Prolonged diarrhea is defined as diarrhea of a presumed infectious cause with acute onset that lasts for at least 7 days; if it lasts for at least 14 days, it is called persistent diarrhea [3]. Although prolonged and persistent diarrhea comprise only a small portion of all childhood diarrhea episodes, they account for more than half of days with diarrhea [4]. Furthermore, extended duration is related to impaired cognitive development, increased risk of malnutrition, micronutrient deficiencies, and death [3], [5], [6].

Reduced host immunity, to a large extent caused by malnutrition and micronutrient deficiencies, plays an important role in the development of persistent diarrhea [3]. Persistent diarrhea is common in populations with a high prevalence of stunting and wasting [7], and administration of zinc during acute diarrhea reduces the duration and the risk of persistence [8]. However, the mechanisms behind it and the relative contribution of different risk factors such as impaired innate or acquired immunity is still unclear [5].

In many areas of the world, children experience repeated episodes of diarrhea [9]. Many cases are brought to physicians or other health workers who may pay little attention to these very common, usually mild but sometimes fatal illnesses. Current WHO management guidelines for acute, uncomplicated diarrhea are advice on home fluids, oral rehydration if the child has signs of some dehydration and continued feeding combined with oral zinc treatment [10]. Unfortunately, antibiotics, which are indicated in special syndromes such as cholera and bacterial or amoebic dysentery, are overused and may be harmful to the individual child. Because diarrhea is so common, such misuse contributes substantially to the spread of antibiotic resistance.

Thus, there is a need to, already in the acute phase, identify children at risk of severe illness beyond the identification of dysentery and dehydration.

## Materials and Methods

We observed 391 episodes of acute non-bloody diarrhea in 335 children 6 to 35 months of age until cessation of the episode, to identify factors that were associated with a duration of>7 days after visiting our study clinic.

**Table 1:** Variables assessed in the initial regression models of 335, 6–35 month old Nepalese children with 391 episodes of acute diarrhea in a study to assess predictors of diarrhea with extended duration.

Age categories	6–11, 12–23, ≥24
Gender	male or female
Whether the child was breastfed	yes or no
Whether the child was exclusively breastfed	yes or no
Stool frequency 24 hours prior to enrollment,	number of stools in 4 categories
Presence of watery stools 24 hours prior to enrollment	yes or no
Fever during episode, reported by caretaker	yes or no
Number of days with diarrhea before clinic visit	1–4
Dehydrated at enrolment*	yes or no
Presence of fever (axillary temperature >38°C)	yes or no
Stunted, defined as being less than 2Z length for age†	yes or no
Wasted, defined as being less than 2Z weight for length†	yes or no
Hemoglobin (g/dL)	hemoglobin level at enrolment
Plasma zinc (µmol/L)	zinc level at enrolment
Recruited from joint families	yes or no
Families owning their own land	yes or no
Families with only one child	yes or no
Families living in only one room	yes or no
Mothers age	years
Any schooling of mother	yes or no
Any schooling of father	yes or no

* Dehydration defined according to WHO guidelines.

† National Center for Health Statisitics, 2000.

The study was conducted in Bhaktapur city near Kathmandu, the capital of Nepal from June 1998 till September 2000 including three wet and hot seasons. We included children with diarrhea enrolled in the placebo limb of a clinical trial measuring the effect of zinc on the outcome of acute diarrhea [11]. Cases of acute diarrhea were identified through weekly household visits and through visits to a field clinic. Study physicians undertook the initial interview and clinical examination, while trained field workers visited the homes for follow-up every fifth day until recovery. There were no known unusual or significant outbreaks of particular pathogens during this period.

The definitions and cut offs used in this analysis were decided before initiation of the study and are the same as those that were used in the analysis of the clinical trial during which the data was generated [11]. Diarrhea was defined as the passage of three or more watery or loose motions and a recent change in stool character in the preceding 24 hours. Only children with acute diarrhea that had lasted for four days or less were enrolled. A diarrhea free day was any day after inclusion with less than three loose and no watery stools. Recovery was defined as two consecutive days without diarrhea. Because the term “Prolonged diarrhea” is now used for episodes lasting 7 days or more, we here define a child as having diarrhea with extended duration when his or her diarrhea lasted longer than 7 days after inclusion. Children were allowed to reenter the study if more than four months had lapsed since the end of the last follow up period. Fifty-four children were included twice and two were included three times.

Acute lower respiratory infections, dysentery, anemia and severe malnutrition were managed according to WHO guidelines [12]. Oral rehydration salts (ORS) packets were given to the caretaker with instructions on its mixing and administration [12]. Whenever caretakers, usually mothers, or field workers suspected that medical care was needed, the children were taken to the clinic for examination by one of the study physicians. The examination and any treatment or transportation to other health facilities were provided free of charge. The study had ethical clearance from the Nepal Health Research Council, Kathmandu, Nepal. The implementation of all aspects of the project was in agreement with the international ethical guidelines for research involving human subjects as stated in the latest version of the Helsinki Declaration. Informed and, when possible, written consent was obtained from at least one of the parents.

The field workers were extensively trained to perform the clinical examination, retraining was done every five months throughout the data collection period [11]. During the entire study, in 8% of all home visits, supervisors or study physicians overlooked the field workers or undertook independent visits, again filling in the field workers' questionnaires in addition to a separate form on field worker performance. This was done to ensure appropriate interaction between the participants and the study staff and to maximize data quality.

The children were weighed to the nearest 100 g and length to the nearest millimeter was measured using a locally made length board. Blood sampling and plasma zinc analysis were undertaken as described previously [11]. We examined the children and obtained day-wise information on illnesses every fifth day until recovery from the diarrheal episode. At each visit, we recorded details of illness characteristics, including the number and character of stools on each day since the last visit. We were not able to obtain the date of recovery from one child because the caretaker withdrew her consent and in another because he was absent during the scheduled visits.

All forms were checked manually by supervisors and physicians for completeness and consistency. The data was then double entered into databases with computerized logic, range and consistency checks. If errors were detected, the forms were returned to the field for correction the next working day. Weight for age, length for age, and weight for length z-scores were calculated using LMS values obtained from CDC growth charts. A wasted child had a weight for length Z –score <-2, while a stunted child had a length for age Z-score <-2. Statistical analyses were undertaken using Stata®, version 10 (StataCorp, College Station, TX) and R version 2.0.0 (The R Foundation for Statistical Computing).

We assessed the association of relevant independent variables with the outcome in multiple logistic regression models. We used generalized additive models [13] to assess the relationship between the logit of the outcome variable and the continuous covariates. Adjustment for repeated entry of the same child was done using generalized estimation equations (GEE) [14] with an exchangeable covariance structure. The variables that were included in the initial crude assessments are shown in Table 1. Based on these estimates, we selected variables for the multivariable model, as described elsewhere [15]. A number of possible interactions between independent variables were also assessed (breastfeeding×age, breastfeeding×stool frequency, age×season, and stool frequency×age). In an alternative procedure, we explored every possible combination of independent variables. For each combination, the Akaike's information criterion (AIC) value was computed [16], and the model with the lowest value selected. Crude and adjusted odds ratios (ORs) are shown; the variables that were not retained in the multivariable models were left out. A *P*–value of less than 0.05 was considered to reflect statistical significance.

## Results

The baseline characteristics of the study participants are listed in Table 2. Forty-two percent of the 391 cases of acute diarrhea were included during infancy and there were more boys (55%) than girls. Eighty-two percent were breastfed, 4.3% exclusively so. Thirty percent were stunted and 24% wasted. The residence of the children was evenly distributed among the 17 administrative areas of Bhaktapur municipality. Twelve percent of the children had some dehydration at enrolment, while no cases presented with severe dehydration.

**Table 2:** Baseline characteristics of 335, 6–35 month old Nepalese children with 391 episodes of acute diarrhea in a study to assess predictors of diarrhea with extended duration.

	n (%)	Mean±SD
Age in months		15.4±7.8
6–11 months	164 (41.7)	
12–23 months	153 (38.9)	
24–35 months	76 (19.3)	
Male	216 (55.0)	
Breastfed	324 (82.4)	
Exclusively breastfed	17 (4.3)	
Total number of stools 24 hours prior to enrollment		8.7±4.0
Fever during episode, reported by caretaker	191 (48.6)	
Dehydrated at enrolment*	50 (12.7)	
Stunted, defined as being less than 2Z length for age†	119 (30.3)	
Wasted, defined as being less than 2Z weight for length†	93 (23.7)	
Hemoglobin (g/dL)		11.2±1.1
Plasma zinc (µmol/L)		8.3±1.9

Dehydration defined according to WHO guidelines.

National Center for Health Statisitics, 2000.

Sixty-seven (17.1%) of the 391 diarrhea cases for whom we had complete follow-up information had recovered one day after enrolment, 250 (63.9%) recovered within three days, 336 (85.9%) had an episode that lasted for a week or less, and 55 (14.1%) lasted for > 7 days after enrolling into the study. Nineteen (4.9%) episodes had a total duration of at least 14 days and could be classified as persistent diarrhea.

Identical variables were selected by the two variable selection procedures for building the final multivariable logistic regression model for extended illness duration. Breastfeeding status, age, stool frequency and season were all independently associated with an increased risk of having this outcome (Table 3). However, stunting, wasting, underweight, fever, history of watery stools, vomiting and respiratory symptoms, hemoglobin and plasma zinc levels were not. Nor were maternal or paternal education or age, type and size of housing, whether the family of the child owned farming land or other variables reflecting socioeconomic status. Excluding children who were included more than once from the analyses did not alter the ORs substantially, using logistic regression models that did not take clustering into account and GEE models did also result in nearly identical results and revealed the same significant associations.

**Table 3:** Determinants of having a diarrheal episode that lasted for more than 7 days after consultation for acute diarrhea among 6–35 month old Nepalese children, as derived from a multivariable logistic regression model.

	Crude	Adjusted
	OR* (95% CI)	OR* (95% CI)
Age category		
>24 months	1	1
12–23 months	1.4 (0.6, 3.5)	9.9 (2.1, 45.7)
6–11 months	2.1 (0.9, 5.0)	17.0 (3.5, 83.1)
Breastfeeding		
yes	1	1
no	1.4 (0.7, 2.8)	9.3 (2.4, 35.7)
Stool frequency		
<9	1	1
9–12	2.3 (1.2, 4.5)	2.0 (1.0, 4.0)
13–16	3.4 (1.4, 8.6)	2.9 (1.1, 7.6)
>16	7.5 (2.6, 21.9)	8.5 (2.8, 26.1)
Season		
Dry	1	1
Wet	2.1 (1.2, 3.8)	2.6 (1.4, 4.9)

* OR  =  Odds Ratio.

### Logistic regression model with age and stool frequency as continuous variables

**Figure 1:**
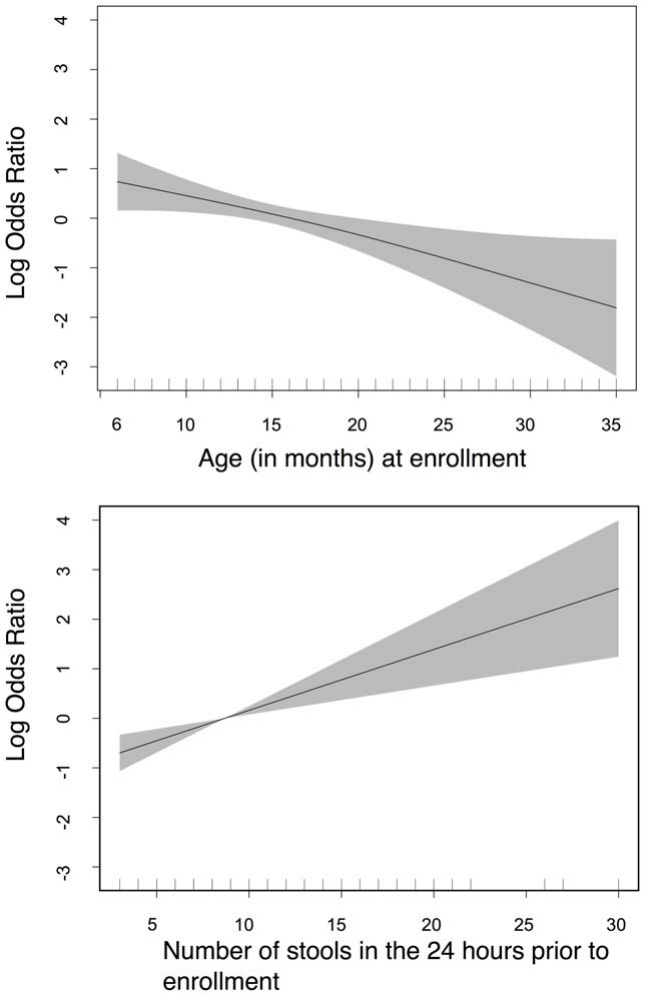
The association between the odds of extended duration of an episode of diarrhea and age and stool frequency in Nepalese children 6 to 35 months of age.

For the final models we categorized stool frequency and age (Table 3). The graphs depicting these relationships using generalized additive models with a logit link function showed that the associations between the logit of the response variable and age or stool frequency are linear (Figure 1). The OR for one unit increment in age (month) was 0.92 (95%CI: 0.87,0.97), and 1.13 (95%CI: 1.06, 1.21) for each additional stool during the last 24 hours. Age was did not modify the effect of breastfeeding [OR for the interaction term = 0.99 (95%CI: 0.87, 1.13)].

### Model assessment

The ability of the logistic regression model to predict whether a child had prolonged illness was acceptable as the area under the receiver operator characteristic curve (AUC) was 0.74. Summary measures of goodness of fit assessed by the method of Hosmer and Lemeshow [17] were also in the acceptable range.

## Discussion

In the present study, we identified independent risk factors for having diarrhea of extended duration.

The odds of prolonged illness was 9.3-fold higher if a child was not breastfed, this effect was not modified by age. This is an argument for recommending breastfeeding, also beyond infancy in populations where childhood diarrhea is common. The benefits of breast milk on child health and survival are well recognized [18], and an increased risk of persistent diarrhea in children that are not breastfed have been demonstrated previously [19]. Sequential infection may be a cause of persistent diarrhea [20] and the protective effect of breastfeeding may be through a reduction of the microbial exposure by a reduced intake of contaminated food or water and/or by immunoglobulins and other bioactive substances and essential nutrients in the breast milk. It should be noted that in our population, only four percent were exclusively breastfed. Children in Nepal are usually introduced to complementary food from four months of age and our children were all aged six months or older.

Younger children are less likely to have had previous exposures to enteropathogens than do older children. Previous exposure to a microorganism may induce specific immunity, which in turn may reduce stool frequency and episode duration. Moreover, the immune system goes through a development throughout early childhood independently of antigen exposure [21]. These two factors may in part explain why children aged 6–11 months had a higher risk of continuation of diarrhea compared to older children in our study.

Zinc is now recommended for the treatment of childhood diarrhea in low-income countries. Oral zinc treatment reduces episode duration and the risk of prolonged illness [11], [22], [23]. Furthermore, administration of zinc is less efficacious in infants compared to in older children [11], [24], [25], and no study that only enrolled children less than 6 months of age has demonstrated a therapeutic effect of zinc [26], [27]. Thus, it seems that zinc is less efficacious in the age group where it is needed most. It should also be noted that the adjusted OR for extended diarrheal duration of not being breastfed was 9.3 (in favor of breastfeeding) while in the same population and for the same outcome, the OR for zinc treatment was 1.8 (in favor of zinc) [11]. Thus, breastfeeding promotion may be a more important intervention to prevent extended diarrheal duration than zinc treatment and may be more effective in infants who are at a substantially higher risk for prolonged and even persistent diarrhea compared to older children.

There was a strong association between the number of stools prior to enrolment and episode duration. This is in line with findings in Peru, where the risk of persistent diarrhea was associated with > 5 diarrheal stools per day in the first week of the acute episode [28]. In fact, in our study, there was a linear relationship between the number of stools and the logit of illness with extended duration. Innate and acquired immunity may reduce the severity and thereby the stool frequency and duration. Thus, stool frequency is probably related to the intensity of the intestinal infectious process. However, presence of fever, reduced general condition, or other clinical signs of severe illness at enrolment were not associated with prolonged disease. Stool frequency may be an indicator of the severity of the infection in the gut that may not be reflected in the other above-mentioned general signs of infection and could, in combination with the other predictors, help to identify children in most need of special attention.

The effect of falling ill in the warm and wet period of the year on the duration of diarrhea may be due to seasonal variations in the transmission of different etiologic agents. During the warmer periods, the bacterial load increases by rapid growth in contaminated foods and possibly water [29] and increases the risk of severe disease. Viral diarrhea, which typically lasts for less than a week, is more prevalent during the cooler months while protozoan agents are more common during the wet and warm seasons [30], [31]. *Cryptosporidium parvum* and *Cyclospora cayetanesis* are associated with long lasting diarrhea and these are encountered more frequently during the wet season [32]. Furthermore, the parents spend more time working in the field in the wet and warm season and may then have less time to care for their children.

Although stunting and wasting were not predictive for duration >7 days, wasting was independently associated with a duration >14 days (data not shown). Wasting represents a state of acute malnutrition, and is also associated with several other serious infections. All children with this condition should accordingly be given special attention with nutritional support, regardless of the risk predicted by age, breast feeding-status, stool frequency and season.

Low plasma zinc is associated with an increased risk of infections [33]. In our study, however, the plasma zinc concentration was not associated with illness duration. Several factors, such as the degree of inflammation and hemodilution, affect plasma zinc levels during infections [34], [35]. Thus, plasma zinc levels may not be a good marker of zinc status when assessed during diarrhea, which could explain the lack of association with illness duration.

We excluded children with a preenrollment duration of more than 4 days. Thus, our findings may not be relevant for children who have had diarrhea for a longer time before visiting our clinic. Furthermore, our study was not powered to measure an impact on persistent diarrhea. Further studies should focus on identifying predictors for both prolonged and persistent diarrhea and identify mechanisms for these conditions.

Oral zinc therapy is recommended to all children in developing countries presenting with diarrhea [10]. However, the risk of prolonged illness is highest in late infancy, these children are also those who are less likely to respond to zinc treatment. Promoting breastfeeding may be the most effective strategy to reduce the incidence of diarrhea of extended duration in infants as well as in young children. A high burden of diarrhea in early childhood may increase the risk for cognitive impairment later in life [36]. Extended diarrhea is also associated with a substantial increased risk of malnutrition as well as persistent diarrhea [4]. Identification of risk factors for long lasting diarrhea and ensuring adequate care for children with these risk factors may thus also have implications beyond the duration of the episode.

## References

[1] Black RE, Cousens S, Johnson HL, Lawn JE, Rudan I (2010). Global, regional, and national causes of child mortality in 2008: a systematic analysis.. Lancet.

[2] Victora CG, Bryce J, Fontaine O, Monasch R (2000). Reducing deaths from diarrhoea through oral rehydration therapy.. Bull World Health Organ.

[3] Black RE (1993). Persistent diarrhea in children of developing countries.. Pediatr-Infect-Dis-J 12: 751–761; discussion.

[4] Moore SR, Lima NL, Soares AM, Oria RB, Pinkerton RC (2010). Prolonged episodes of acute diarrhea reduce growth and increase risk of persistent diarrhea in children.. Gastroenterology.

[5] Bhutta ZA, Nelson EA, Lee WS, Tarr PI, Zablah R (2008). Recent advances and evidence gaps in persistent diarrhea.. J Pediatr Gastroenterol Nutr.

[6] Bhandari N, Bhan MK, Sazawal S (1992). Mortality associated with acute watery diarrhea, dysentery and persistent diarrhea in rural north India.. Acta-Paediatr-Suppl.

[7] Black RE (1993). Epidemiology of diarrhoeal disease: implications for control by vaccines.. Vaccine.

[8] Lazzerini M, Ronfani L (2008). Oral zinc for treating diarrhoea in children..

[9] Black RE, Lopez de Romana G, Brown KH, Bravo N, Bazalar OG (1989). Incidence and etiology of infantile diarrhea and major routes of transmission in Huascar, Peru..

[10] WHO (2004). World Health Organization (WHO) and United Nations Children's Fund (UNICEF)..

[11] Strand TA, Chandyo RK, Bahl R, Sharma PR, Adhikari RK (2002). Effectiveness and efficacy of zinc for the treatment of acute diarrhea in young children.. Pediatrics.

[12] World Health Organization (1997). Integrated management of childhood illness..

[13] Wood SN (2000). Modelling and Smoothing Parameter Estimation with Multiple Quadratic Penalties.. JRStatistSocB.

[14] Diggle PJ, Liang KY, Zeger SL (1994). Analysis of Longitudinal Data..

[15] Hosmer DW, Lemeshow S (2000). Applied Logistic Regression..

[16] Collett D (1994). Modelling Survival Data in Medical Research..

[17] Lemeshow S, Hosmer DW (1982). A review of goodness of fit statistics for use in the development of logistic regression models.. Am J Epidemiol.

[18] Molbak K, Gottschau A, Aaby P, Hojlyng N, Ingholt L (1994). Prolonged breast feeding, diarrhoeal disease, and survival of children in Guinea-Bissau.. BMJ.

[19] Molbak K, Jakobsen M, Sodemann M, Aaby P (1997). Is malnutrition associated with prolonged breastfeeding? [letter].. Int J Epidemiol.

[20] Baqui AH, Black RE, Sack RB, Yunus MD, Siddique AK (1992). Epidemiological and clinical characteristics of acute and persistent diarrhoea in rural Bangladeshi children.. Acta.

[21] MacDonald TT, Spencer J (1990). Ontogeny of the mucosal immune response.. Springer Semin Immunopathol.

[22] Bhutta ZA, Bird SM, Black RE, Brown KH, Gardner JM (2000). Therapeutic effects of oral zinc in acute and persistent diarrhea in children in developing countries: pooled analysis of randomized controlled trials.. Am J Clin Nutr.

[23] Sazawal S, Black RE, Bhan MK, Bhandari N, Sinha A (1995). Zinc supplementation in young children with acute diarrhea in India [see comments].. N-Engl-J-Med.

[24] Sazawal S, Black RE, Ramsan M, Chwaya HM, Dutta A (2007). Effect of zinc supplementation on mortality in children aged 1-48 months: a community-based randomised placebo-controlled trial.. Lancet.

[25] Tielsch JM, Khatry SK, Stoltzfus RJ, Katz J, LeClerq SC (2007). Effect of daily zinc supplementation on child mortality in southern Nepal: a community-based, cluster randomised, placebo-controlled trial.. Lancet.

[26] Brooks WA, Santosham M, Naheed A, Goswami D, Wahed MA (2005). Effect of weekly zinc supplements on incidence of pneumonia and diarrhoea in children younger than 2 years in an urban, low-income population in Bangladesh: randomised controlled trial.. Lancet.

[27] Fischer Walker CL, Bhutta ZA, Bhandari N, Teka T, Shahid F (2006). Zinc supplementation for the treatment of diarrhea in infants in Pakistan, India and Ethiopia.. J Pediatr Gastroenterol Nutr.

[28] Lanata CF, Black RE (1991). Lot quality assurance sampling techniques in health surveys in developing countries: advantages and current constraints.. World Health Stat Q.

[29] Rowland HA (1978). The pathogenesis of diarrhoea.. Trans R Soc Trop Med Hyg.

[30] Cook Mills JM, Wirth JJ, Fraker PJ (1990). Possible roles for zinc in destruction of Trypanosoma cruzi by toxic oxygen metabolites produced by mononuclear pha0067ocytes.. Adv Exp Med Biol.

[31] Molbak K, Hojlyng N, Gottschau A, Sa JC, Ingholt L (1993). Cryptosporidiosis in infancy and childhood mortality in Guinea Bissau, west Africa.. BMJ.

[32] Perch M, Sodemann M, Jakobsen MS, Valentiner-Branth P, Steinsland H (2001). Seven years' experience with Cryptosporidium parvum in Guinea-Bissau, West Africa.. Ann Trop Paediatr.

[33] Bahl R, Bhandari N, Hambidge KM, Bhan MK (1998). Plasma zinc as a predictor of diarrheal and respiratory morbidity in children in an urban slum setting.. Am J Clin Nutr.

[34] Brown KH (1998). Effect of infections on plasma zinc concentration and implications for zinc status assessment in low-income countries.. Am J Clin Nutr.

[35] Strand TA, Adhikari RK, Chandyo RK, Sharma PR, Sommerfelt H (2004). Predictors of plasma zinc concentrations in children with acute diarrhea.. Am J Clin Nutr.

[36] Walker SP, Wachs TD, Gardner JM, Lozoff B, Wasserman GA (2007). Child development: risk factors for adverse outcomes in developing countries.. Lancet.

